# LPBF Processability of NiTiHf Alloys: Systematic Modeling and Single-Track Studies

**DOI:** 10.3390/ma17164150

**Published:** 2024-08-22

**Authors:** Hediyeh Dabbaghi, Mohammad Pourshams, Mohammadreza Nematollahi, Behrang Poorganji, Michael M. Kirka, Scott Smith, Chins Chinnasamy, Mohammad Elahinia

**Affiliations:** 1 Department of Mechanical, Manufacturing, and Industrial Engineering, University of Toledo, Toledo, OH 43606, USA; hdabbag@rockets.utoledo.edu (H.D.); mohammad.pourshams@utoledo.edu (M.P.); m.nematolahi@gmail.com (M.N.); behrang.poorganji@utoledo.edu (B.P.); 2 Oak Ridge National Laboratory, Manufacturing Science Division, Energy Science and Technology Directorate, Oak Ridge, TN 37830, USA; kirkamm@ornl.gov (M.M.K.); smithss@ornl.gov (S.S.); chinnasamyc@ornl.gov (C.C.)

**Keywords:** high-temperature shape memory alloys (HTSMAs), laser powder bed fusion (LPBF), solidification cracking, processability assessment, computational modeling, laser remelting experiments

## Abstract

Research into the processability of NiTiHf high-temperature shape memory alloys (HTSMAs) via laser powder bed fusion (LPBF) is limited; nevertheless, these alloys show promise for applications in extreme environments. This study aims to address this limitation by investigating the printability of four NiTiHf alloys with varying Hf content (1, 2, 15, and 20 at. %) to assess their suitability for LPBF applications. Solidification cracking is one of the main limiting factors in LPBF processes, which occurs during the final stage of solidification. To investigate the effect of alloy composition on printability, this study focuses on this defect via a combination of computational modeling and experimental validation. To this end, solidification cracking susceptibility is calculated as Kou’s index and Scheil–Gulliver model, implemented in Thermo-Calc/2022a software. An innovative powder-free experimental method through laser remelting was conducted on bare NiTiHf ingots to validate the parameter impacts of the LPBF process. The result is the processability window with no cracking likelihood under diverse LPBF conditions, including laser power and scan speed. This comprehensive investigation enhances our understanding of the processability challenges and opportunities for NiTiHf HTSMAs in advanced engineering applications.

## 1. Introduction

High-temperature shape memory alloys (HTSMAs) exhibit remarkable properties, including substantial reversible shape changes under high-stress conditions, facilitated by martensitic transformations above 100 °C [1]. Among HTSMAs, NiTi-based alloys have attracted considerable attention for their versatility in high-temperature solid-state actuation across various industries, such as automotive and aerospace industries [1,2,3,4]. Ternary HTSMAs, incorporating elements like Pt, Pd, Au, and Hf, along with NiTi, have emerged as promising candidates [5,6,7]. Research indicates that incorporating Hf has a more significant effect on transformation temperatures compared to Pd and Au [1,3]. NiTiHf alloys offer broader applicability in high-temperature applications compared to costly alternatives, such as NiTiAu, NiTiPd, and NiTiPt [8,9]. Previous studies highlight the influence of composition —whether they tend to be Ti-rich or Ni-rich—and heat treatment methods on precipitate properties in NiTiHf alloys, emphasizing the complexity of alloy design [10]. While earlier research has focused on (Ti + Hf)-rich compositions, recent investigations reveal challenges in stoichiometric or Ti-rich alloys with lower Hf content, as well as those with higher Hf additions [11,12,13]. These challenges include thermal cyclic response degradation, brittleness, and poor thermomechanical behavior [1,14,15,16]. In contrast, in Ni-rich counterpart shape memory alloys (SMAs), precipitation strengthening has been demonstrated to effectively enhance the shape memory properties of Ni-rich NiTiHf alloys [17,18,19,20]. The formation of nanoscale H-phase precipitates [21,22,23] enhances both the yield strength and the shape memory behavior of the alloy [20]. Therefore, recent exploration has shifted towards Ni-rich NiTiHf alloys. Initial findings on a Ni50.3Ti20Hf alloy suggest improved shape memory behavior and excellent superelastic properties [11]. Meng et al. demonstrated precipitate formation in Ni-rich Ni50.3Ti20Hf alloys, leading to increased transformation temperatures [24,25]. Further studies by Benafan et al. revealed impressive mechanical and functional properties of Ni-rich Ni50.3Ti29.7Hf20 alloys, including near-perfect superelasticity and excellent dimensional stability [26]. Evirgen et al. investigated the impact of precipitation on a Ni50.3TiHf15 alloy, observing changes in transformation temperatures and strain recovery [27].

Conventional techniques like arc melting [28], vacuum induction melting [20], and plasma arc melting [29] are commonly used to manufacture NiTiHf alloys, spanning Hf concentrations of 1 to 50 at. % [9,28]. However, additive manufacturing (AM), notably laser powder bed fusion (LPBF), has emerged as a promising alternative, which offers intricate geometry creation and customization of microstructures, compositions, and thermomechanical properties [30]. This adaptability is especially valuable, allowing for customization beyond conventional methods [31,32,33]. Recently, numerous studies have focused on investigating the processability of Ni-rich Ni50.4TiHf20 (at. %) HTSMAs using LPBF. These investigations thoroughly examined aspects such as processing parameters and defects, microstructural characteristics, phase transformations, thermomechanical behaviors, mechanical properties, and oxidation kinetics [34,35,36,37,38].

The selection of materials and processing parameters is important for the success of laser powder bed fusion (LPBF), significantly affecting the accuracy of parts and metallurgical properties. Tailoring alloys, particularly NiTiHf with varied Hf content, show promise in improving processability and printability [39]. The LPBF AM process poses significant challenges due to exposure to large thermal gradients, intricate thermal histories, and rapid solidification conditions [40]. These unique conditions often result in printed parts with differing microstructures, properties, and performance compared to conventional methods [41]. In LPBF, discrepancies between processing parameters and alloy characteristics can result in a range of challenges, including residual internal stresses that lead to distortions and cracking, nonuniform melting and solidification that result in hot tearing or cracking, vaporization of alloying components that cause undesired stoichiometry, unintentional gas entrapment (known as keyholing), and incomplete wetting and spreading that lead to inadequate layer fusion and balling [42].

In LPBF, solidification cracking, also known as hot cracking, poses a critical challenge during the final stages of the solidification process [43]. This phenomenon occurs primarily in the partially solid state, resulting in irreversible cracks [44,45]. It is driven by thermal stresses induced by solidification shrinkage as the lack of compensation from liquid flow causes the material to fracture [46]. Initiation sites for cracks often form above the solidus temperature, particularly beyond that of the interdendritic region, where cavities and pores develop [47]. The alloy’s ability to fill the semisolid zone with liquid metal influences the susceptibility to solidification cracking. The width of this semisolid mush, determined by the solidification range—spanning between the liquidus and solidus temperatures—is crucial in evaluating crack susceptibility [48]. A narrower solidification range allows for more rapid traversal of the highly susceptible microstructure by temperature changes [49]. Research by Shankar et al. [50] demonstrated a substantial reduction in the hot cracking density of stainless steel, from 1.1 to 0.1 mm/mm^2^, by narrowing the solidification range from 68 to 21 °C. Other models, such as those integrating liquid feeding (0–90% solid) and liquid film/droplet transformation (0–94% solid) [43], adjust the critical solidification range, often in terms of solidification time. Additionally, Clyne and Davis [51] proposed assessing hot cracking sensitivity based on the transition time of the mushy zone from liquid to solidus. Overall, narrowing the solidification range, often achieved by increasing the solidus temperature, is crucial for reducing the susceptibility to solidification cracking.

Various models have been developed to assess solidification cracking susceptibility [52,53,54,55,56,57,58]. Notably, the Kou model [59,60] stands out for its comprehensive approach, particularly in evaluating solidification cracking in fusion welds. This model provides valuable insights by correlating solidification gradient with material susceptibility. Importantly, the Kou model is closely associated with the Scheil-Gulliver model [61], commonly used for predicting solidification gradients. Recent advancements include the utilization of the DICTRA (diffusion-controlled transformations) package within Thermo-Calc software, which offers a robust method for modeling solidification gradients considering thermal history and kinetics [62]. Additionally, the Scheil solidification model, employing the Gulliver-Scheil equation [48,63], has proven effective in anticipating crack susceptibility across various aluminum, magnesium, and nickel-based alloys [59,60,64,65,66,67]. While welding and Additive Manufacturing (AM) share similarities, the utilization of the Kou model to evaluate solidification cracking susceptibility in AM processes is underexplored. Limited studies have investigated this aspect for various materials processed using laser powder bed fusion (LPBF), including Fe-based alloys [68,69], Ni-based superalloys [70,71,72,73], and Al alloys [74,75,76]. Numerous studies have emphasized the critical role played by both computational simulations and experimental validation in the prediction of cracking and the evaluation of alloy processability, ensuring comprehensive and reliable insights [77]. The processability of high-alloyed tool steels within LPBF [78] often results in cracking despite achieving densification. Increased carbon content correlates with decreased processability and compromised integrity due to heightened susceptibility to hot cracking, influenced by factors like wide solidification intervals ΔT. Analysis of a CoNi-based superalloy’s cracking behavior in relation to carbon and boron content [79] reveals solidification cracking predominance, mainly parallel to the build direction, aligning with Kou’s criteria for additive manufacturing of superalloys to prevent microcracking. Investigating IN738LC superalloy’s crack susceptibility through varying volume energy densities (VEDs) via computational simulation [80] highlights predominantly solidification-type cracks, particularly at grain boundaries with higher misorientation.

Although additive manufacturing typically involves creating complete 3D parts, single laser track experiments, both with and without powder, provide critical insights into processability and printability. These experiments act as a screening tool for identifying defect susceptibility in laser processing and manufacturing, particularly in laser powder bed fusion (LPBF). Single-track experiments help elucidate the effects of laser parameters—such as power, speed, and focus—on melt pool geometry, solidification behavior, and microstructural evolution. These factors are essential for optimizing the LPBF process, as they directly influence the mechanical properties and performance of the final bulk material. Moreover, analyzing crack behavior through single-track experiments is crucial, as it is closely linked to processing parameters and microstructural evolution during melt pool formation [81,82]. High-strength aluminum alloy AA2024’s printability for LPBF, compared to AlSi10Mg, was assessed, and LPBF-induced solidification cracking in AA2024 was studied through single-track experiments [74]. Seulbi Lee et al. conducted a comprehensive analysis of single-track behavior, identifying different types of cracks, including longitudinal and transverse cracks, which were classified as solidification and thermal cracks [83]. Mohammadpour et al. combined single-track experiments and thermodynamic simulations to study the microstructure of as-built IN625, achieving a close simulation-experiment agreement [84,85]. Ghosh et al. investigated Inconel 625 single tracks without powder, validating FEM simulations and proposing a method to assess crystal shape formation based on temperature and solidification rate [86].

To date, limited studies have explored cracking susceptibility in NiTiHf alloys through LPBF systems. Nematollahi et al. [35] conducted a comprehensive investigation by fabricating Ni-rich Ni50.4TiHf20 alloys using LPBF with various processing parameters. They found that lower energy densities yielded fewer defects, while higher densities induced long cracks due to excessive residual stress. Despite these valuable findings, a broader spectrum of NiTiHf alloys with Hf compositions ranging from 1 to 20 at. % remains relatively unexplored in LPBF. Thus, our study aims to extend this research by investigating the printability of NiTiHf alloys with four distinct Hf contents: 1%, 2%, 15%, and 20%. Using NiTi${Hf}_{20}$ as a reference will provide insights into LPBF effects on different alloy compositions. Our objective is to identify alloys with optimal processability and printability. We evaluate the printability of these alloys using computational and experimental approaches, focusing on predicting solidification cracking likelihood under various process conditions such as laser power and scan speed to define an optimal processing window [87,88,89,90]. Initial assessments of solidification cracking susceptibility rely on the analysis of Kou’s index, complemented by solidification gradient calculations using the Scheil–Gulliver model implemented in Thermo-Calc. Additionally, due to the significant cost and limited availability of Hf powder [91,92], laser remelting experiments explore parameter impacts on different NiTiHf ingots in LPBF processing. This involves printing single tracks within the LPBF machine to validate computational results and establish an effective framework for evaluating printability without using powder. Cracks in single tracks indicate the alloy’s unsuitability for processing, leading to its exclusion from further consideration when employing powder.

## 2. Computational Method

Four series of NiTiHf ingots, namely, as-cast ${Ni}_{50.3}Ti{Hf}_{1}$ (at. %) and ${Ni}_{50.3}Ti{Hf}_{2}$ (at. %) and as-extruded ${Ni}_{50}Ti{Hf}_{15}$ (at. %) and ${Ni}_{50.3}Ti{Hf}_{20}$ (at. %), were analyzed to evaluate their printability. This assessment was based on their solidification characteristics, determined using the calculation of phase diagrams (CALPHAD) methodology. To achieve this, the following approaches were implemented:

### 2.1. Solidification Temperature Range (STR)

The solidification temperature range (STR) is considered a standalone index in assessing the susceptibility of alloys to cracking during solidification. It is utilized as an independent measure to evaluate the likelihood of alloys to undergo solidification cracking, with higher solidification ranges correlating to increased vulnerability to such phenomena. Essentially, the STR defines the temperature range between the liquidus and solidus points, covering the entirety of the solidification process [49].

In this study, the STR is employed as a means of assessing the susceptibility of alloys to solidification cracking. A wider solidification range signifies a greater likelihood of encountering solidification-induced cracks. By calculating the solidification range for each of the four alloys under investigation, a comparative analysis is conducted to determine the alloy with the least susceptibility to cracking during solidification.

### 2.2. Solidification Simulation and Cracking Susceptibility Index (Kou’s Criterion)

The solidification gradient and cracking susceptibility were evaluated using the Scheil–Gulliver solidification model [93] in Thermo-Calc/2022a with TCNI12: Ni-Alloys v11.0 data base, a commercial software for calculating thermodynamic phase diagrams (CALPHAD) [94]. Thermo-Calc’s Property Modeling module can be used to calculate various properties, including liquidus and solidus temperatures, based on equilibrium phase diagrams. In this study, these temperatures and solidification ranges were determined for the four NiTiHf ingots using Thermo-Calc and the Python-Thermo-Calc module for enhanced precision.

Furthermore, a crack solidification susceptibility index (CSSI) was calculated to predict the susceptibility of the four NiTiHf alloys to solidification cracking based on Kou’s cracking index, building on the work of Clyne and Davies [95]. Kou’s index, validated through experiments, incorporates factors such as the phase diagram, solidification shrinkage, strain rate, cooling rate, and liquid feeding. According to Kou’s methodology, increasing the calculated index near (fs)1/2 = 1 indicates higher susceptibility to cracking by decreasing the growth rate needed for grains to bond together and resist cracking. Higher values also signify an increase in the length of the liquid channel along the grain boundary, hindering the liquid feeding required to fill the grain boundary and resist cracking. From a mechanics perspective, tension facilitates the opening of longer grain boundary channels, thereby promoting easier propagation of cracks.

(1) Kou index=dTdfs12nearfs12=1

Kou’s index is calculated as the maximum value of Equation (1), where T is the temperature and $f_{s}$ is the mole fraction of the solid during solidification. To find Kou’s index, the Scheil solidification calculation was performed using Thermo-Calc. Subsequently, the data on the corresponding temperature-mole fraction of the solid were extracted from Thermo-Calc. The extracted data were then processed using MATLAB/R2022a. The square root of the mole fraction of the solid was calculated, and the steepness of the temperature square root of the mole fraction of the solid curve was determined near (f_s_)^1/2^ = 1, which represents the maximum value of |dT/d((f_s_)^1/2^)|, i.e., Kou’s index.

## 3. Experimental Method

Stoichiometric ${Ni}_{50.3}Ti{Hf}_{1}$, ${Ni}_{50.3}Ti{Hf}_{2}$, ${Ni}_{50}Ti{Hf}_{15}$, and ${Ni}_{50.3}Ti{Hf}_{20}$ (at. %) ingots were used as the base plate. The first three ingots were vacuum-induction-melted (VIM) in a graphite crucible and cast into a 25.4 mm diameter by 102 mm long copper mold and then vacuum-homogenized at 1050 °C for 72 h. The ${Ni}_{50.3}Ti{Hf}_{20}$ ingot was vacuum-induction-skull-melted (VISM) using a segmented water-cooled copper hearth, poured on a 3” steel mold, followed by vacuum arc remelting (VAR) into a 6.7” ingot, and then homogenized at 1050 °C for 72 h. The chemical composition of the alloys is shown in Table 1. Bulk chemical composition was determined using a SPECTRO Across MV inductively coupled plasma atomic emission spectrometer (ICP-AES) for detecting metallic elements, and the LECO (ON 736 and CS 844 Combustion Analyzers) was used to determine oxygen, nitrogen, and carbon content.

Differential scanning calorimetry (DSC) analysis was carried out using a DSC 250 instrument (TA Instruments (New Castle, DE, USA)), employing a heating/cooling rate of 10 °C/min. DSC serves as a widely accepted technique for characterizing phase transitions in materials, relying on the measurement of heat flow associated with these transitions as a function of temperature. To minimize oxidation effects on the sample, the analysis was conducted under a specified atmosphere, specifically nitrogen. Each single track underwent two thermal cycles between −180 °C and 250 °C to ensure comprehensive characterization of phase transitions. The capability to undergo phase transformation at very low temperatures is particularly advantageous for applications in subzero environments. These properties significantly enhance the material’s functionality and performance, making NiTiHf alloys suitable for a range of applications that operate in cold conditions. Prior to analysis, meticulous sample preparation of all NiTiHf ingots was performed to ensure uniformity and reproducibility of results.

Laser remelting processes were carried out utilizing the LPBF machine (Phenix Systems PXM-3D Systems, Riom, France). With a laser beam diameter of 80 µm and a fiber laser wavelength of 1070 nm, the fabrication process was meticulously conducted within an argon atmosphere to mitigate oxidation effects, ensuring oxygen levels remained below 500 ppm. Following this, precise single-track laser remelting experiments without the addition of powder were directly conducted on the surfaces of ${NiTiHf}_{1}$, ${NiTiHf}_{2}$, ${NiTiHf}_{15}$, and ${NiTiHf}_{20}$ (at. %) ingots. Each track, extending 18 mm in length, maintained a consistent 0.75 mm gap between consecutive tracks.

Following laser processing, each single track was subject to optical microscopy (OM) analysis using a Keyence VHX 6000 microscope to capture top-view images. Substrates containing the single tracks were then cut perpendicular to the track length using a wire electrical discharge machine (EDM) (CUT E 350-George Fischer (GF) machine solution, Schaffhausen, Switzerland) and embedded in epoxy resin in the build direction for cross-sectional analysis. Metallographic polishing was performed until a 1 µm diamond finish was achieved. Subsequent imaging of melt pool depth and width was conducted using an optical microscope (VHX 6000-Keyence, Osaka, Japan).

## 4. Results and Discussion

### 4.1. Solidification Simulation by Thermo-Calc

**Figure 1:**
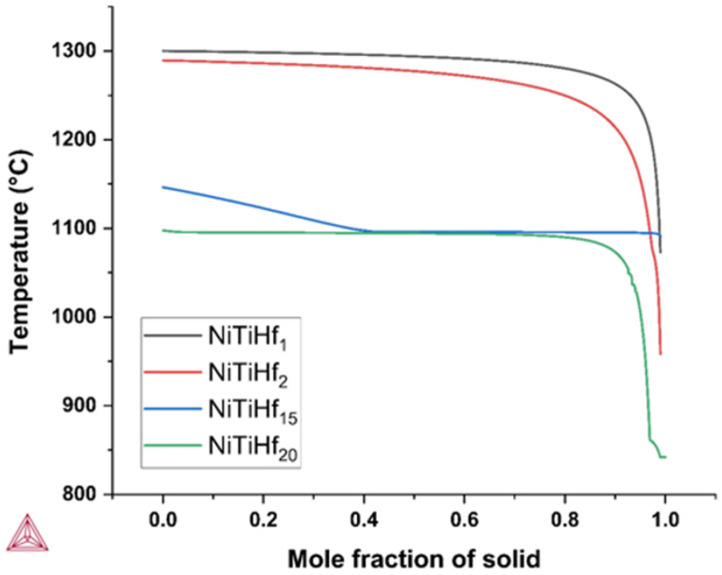
The solidification path calculated using Thermo-Calc of NiTiHf alloys.

The solidification simulation of four NiTiHf ingots with different Hf content was calculated by Thermo-Calc. The results of these simulations are illustrated in Figure 1, displaying the fraction of solids as a function of temperature, based on a Scheil simulation. It is evident that the solidification behaviors differ among the alloys, ultimately impacting their printability. Among the examined alloys, ${NiTiHf}_{2}$ exhibited the highest solidification range, while ${NiTiHf}_{15}$ had the lowest solidification range. Previous studies have suggested that solidification cracking in LPBF is influenced by the solidification range ($∆T=T_{l}-T_{s})$ [96,97]. A wider solidification range leads to extended mushy zones and higher thermal gradients, which increase the risk of cracking due to prolonged shrinkage strain and the formation of weak and low melting point phases [40,98]. Table 2 provides an overview of the phase transition temperatures, including the liquidus, solidus, and thermodynamic simulation for the four NiTiHf alloys. The measured solidification ranges varied between 55.09 °C and 331.24 °C.

For ${NiTiHf}_{1}$, the liquidus temperature initiates at 1300.14 °C without displaying any slope. Throughout the solidification process, the alloy undergoes a transition from a liquid phase to a mixture of liquid and B_2_ phase until reaching a solid mole fraction of 90%. The solidus temperature is observed at 1073.05 °C, when the solid fraction reaches 98%, with the predominant phase being austenite. ${NiTiHf}_{2}$ demonstrates the widest solidification range among the examined alloys. Its liquidus initiates at 1289.57 °C, marked by the light blue region, where the alloy consists of a combination of liquid and austenite B_2_ phases. As the solid mole fraction increases, the alloy undergoes a phase transition, with the solidus temperature observed at 958.33 °C. ${NiTiHf}_{15}$ exhibits a distinct solidification behavior, characterized by a nearly linear slope. Solidification starts at 1146.44 °C, corresponding to the liquidus, and ends at 1091.35 °C. The higher solidus temperatures obtained under equilibrium conditions result in a smaller ∆T characteristic of ${NiTiHf}_{15}$. Following ${NiTiHf}_{2}$, ${NiTiHf}_{20}$ exhibits a considerably broader solidification range, up to 255.92 °C. The solidification process begins at 1097.78 °C, corresponding to the liquidus temperature, and ends at a solidus temperature of 841.86 °C. Throughout this phase, the primary B_2_ phase remains predominant. It is noteworthy that all three alloys, except ${NiTiHf}_{15}$, display markedly steeper slopes, suggesting distinct solidification behaviors and highlighting their diverse thermodynamic properties.

### 4.2. Crack Susceptibility Prediction

**Figure 2:**
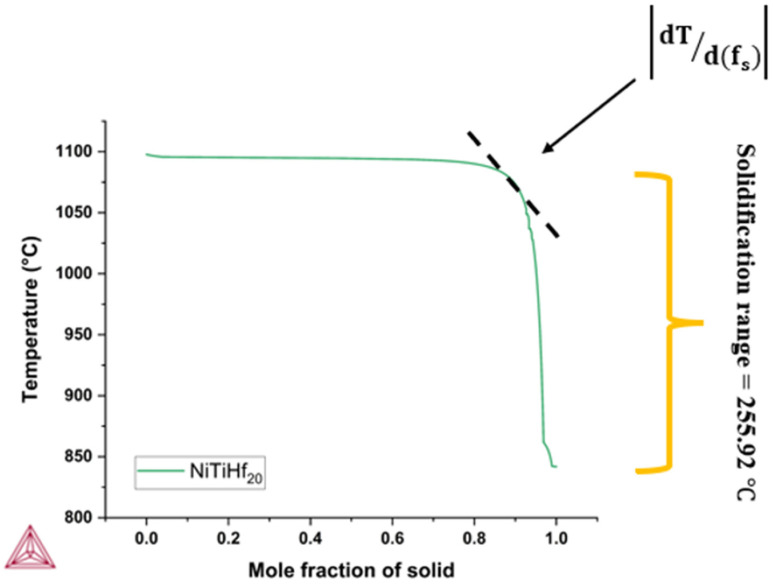
The solidification path calculated using Thermo-Calc of NiTiHf_20_ alloy.

Kou [59] proposed a criterion that links the solidification cracking susceptibility of an alloy with the slope of the T (temperature) vs. ${f_{s}}^{1/2}$ curve. Figure 2 shows the T − ${f_{s}}^{1/2}$ curve for ${NiTiHf}_{20}$ as a representative example. Similar curves were calculated for all other NiTiHf alloys. Kou’s index quantifies this phenomenon by analyzing the steepness of the T − ${f_{s}}^{1/2}$ curve, which represents the relationship between temperature (T) and the square root of the fraction solid ${\left(f_{s}\right)}^{1/2}$ during solidification. The steepness of this curve near the end of solidification (${\left(f_{s}\right)}^{1/2}$ near 1) is directly related to the width of the mushy zone, shown as a dashed black line. Among these four alloys, ${NiTiHf}_{20}$ exhibited maximum steepness of the curve, indicating a higher susceptibility to solidification cracking. Comparing Kou’s index values for different NiTiHf alloys, as shown in Table 3, it can be seen that NiTiHf_20_ is more prone to solidification cracking due to the narrower mushy zone during the final stage of solidification.

Kou’s index values (Table 3) showed that the ${NiTiHf}_{15}$ alloy had the lowest value of 0.0058 among the investigated NiTiHf alloys, indicating its superior resistance to solidification cracking. A lower Kou’s index reflects a reduced tendency for cracking during the manufacturing process. NiTiHf_15_ exhibited a notably low Kou’s index, suggesting enhanced resistance to solidification cracking compared to the other alloys studied. This is due to its favorable solidification behavior, which includes a wider mushy zone, better liquid feeding capabilities, and reduced strain accumulation during the final stages of solidification.

Table 4 presents the solidification temperature range (STR), a normalized index for solidification across all NiTiHf alloys. The calculation was standardized based on the alloy with the highest solidification range, i.e., NiTi${Hf}_{2}$, at 331.24. Subsequently, other alloys were normalized relative to NiTi${Hf}_{2}$ for comparison.

The STR simplifies the assessment of cracking susceptibility, where higher values indicate a greater risk of cracking. As a result, NiTi${Hf}_{2}$ demonstrates the highest susceptibility, followed by NiTi${Hf}_{20}$, NiTi${Hf}_{1}$, and NiTi${Hf}_{15}$, with the latter displaying the lowest susceptibility. The low susceptibility of NiTi${Hf}_{15}$ suggests favorable printability and processability. Additionally, Table 4 outlines Kou’s index, representing the steepness criterion (SC) for cracking susceptibility. Normalization based on this alloy reveals NiTi${Hf}_{20}$ as the one that is most susceptible to cracking, followed by NiTi${Hf}_{1}$, NiTi${Hf}_{2}$, and NiTi${Hf}_{15}$, with the latter having the lowest Kou’s index value of 0.003. Thus, both the STR and SC (Kou) results highlight the low susceptibility of NiTi${Hf}_{15}$ to cracking. However, the ranking of cracking susceptibility among the other three NiTiHf alloys differs. While STR offers simplicity, Kou’s index provides reliable insights, particularly for computational modeling. Validation of the index will be conducted using experimental data from printed NiTiHf single tracks, as detailed in subsequent sections.

## 5. Comparison to Experimental Data

### 5.1. NiTiHf Substrates Characterization

**Figure 3:**
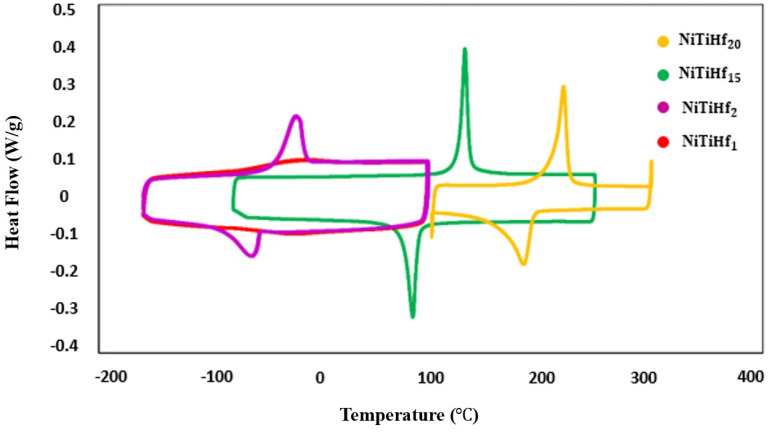
The phase transformation behavior of the NiTiHf alloys captured using DSC characterization.

In order to determine the TTS of four NiTiHf alloys, DSC was utilized with a nitrogen gas atmosphere at a heating rate of 10 °C/min. The phase transformation temperatures, such as martensite finish (M_f_), martensite start (M_s_), austenite start (A_s_), and austenite finish (A_f_), were determined using the tangent method applied to the DSC curve according to ASTM F2004-17 guidelines. The obtained DSC results are depicted in Figure 3, and their corresponding thermodynamic temperatures are listed in Table 5. The analysis of the DSC curve confirms the alloy’s suitability for high-temperature applications.

The transformation temperatures (TTs) of the alloys show a significant increase with higher Hf content in the NiTi alloy. This observation is consistent with the findings of Sanjabi [99], and Tong et al. [100], who reported that transformation temperatures in NiTiHf alloys shift to higher temperatures with increasing Hf composition, rather than Ti content. Conversely, Benafan et al. [101] found that increasing the Ni content from 50 to 51 at. % in ${NiTiHf}_{20}$ alloys resulted in decreased TTs. Furthermore, Umale et al. [102] observed a broad range of transformation temperatures (TTs) from −170 to 500 °C, indicating the significant variability achievable by adjusting the composition in NiTiHf alloys. The transformation temperatures (TTs) in NiTiHf alloys, as depicted in Figure 3, exhibit a notable dependence on composition. Specifically, for Ni-rich compositions, the martensite start temperature (M_s_) initially decreases with increasing Hf content, reaching a minimum before gradually increasing with further Hf addition. For instance, at a Ni content of 50.3 at. %, Ms decreased from 18 °C for 1 at. % Hf to −51 °C for 2 at. % Hf, where the minimum was observed. This trend is consistent with previous findings on Ni-rich compositions [102]. Comparing the DSC results of Ni50.3TiHf2 and Ni50TiHf15, reducing the Ni content from 50.3 at. % to 50 at. % while increasing the Hf content from 2 at. % to 15 at. % led to an increase in M_s_, as shown in Figure 3. Table 5 presents the Hf dependence of the transformation hysteresis (A_f_ − M_s_) for the four NiTiHf alloys. The observed trend of hysteresis variation, initially decreasing and then increasing, corresponded with the findings of Benafan et al. [101], where the Hf content increased from 1 to 2 at. %. The elevated TTs contribute to a more stable microstructure during the printing process, thereby potentially improving print quality.

### 5.2. Single-Track Analysis

#### Top-View Analysis

The printing process in LPBF is significantly affected by the settings of laser power and scanning speed. These parameters are essential in shaping the melt pool, thus affecting the geometry and stability of the printed material. Single tracks were fabricated on a bare substrate via laser remelting, as no powder was utilized in this study, to assess the impact of different combinations of laser power and scanning speed. The processing parameters, along with their linear energy density ($E_{l}=P/v$), are detailed in Table 6.

**Figure 4:**
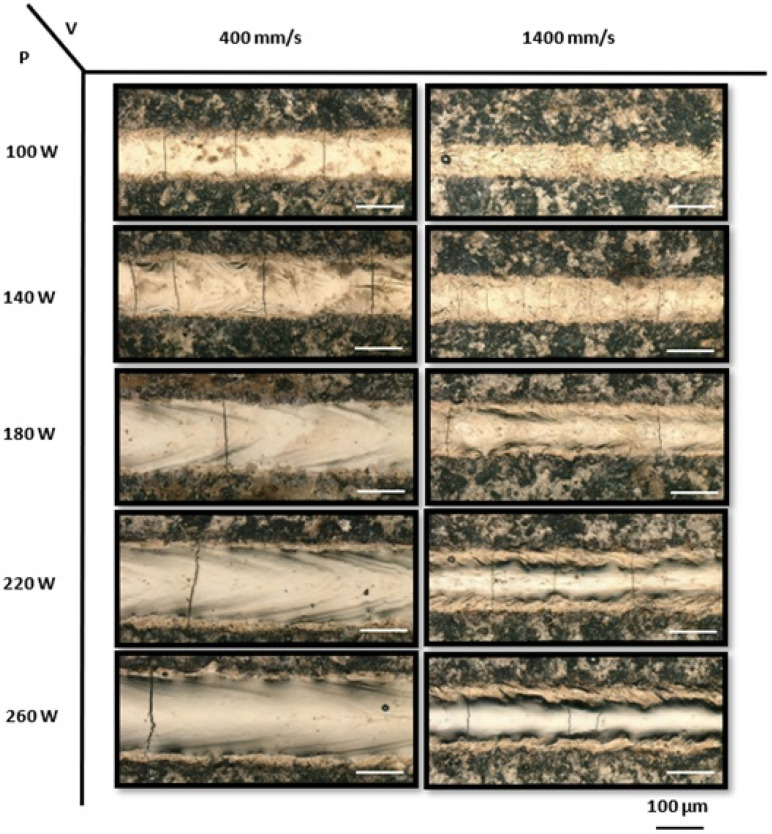
The optical micrographs of the top view of printed NiTiHf1 single tracks with laser powers ranging from 100 to 260 W and scanning speeds of 400 mm/s and 1400 mm/s.

To evaluate the processability of four distinct NiTiHf alloys, a systematic exploration of laser power and scanning speed combinations was conducted. Specifically, for the low-temperature alloys, i.e., ${NiTiHf}_{1}$ and ${NiTiHf}_{2}$, a total of 30 single tracks were printed. Laser powers varied from 100 to 260 W, while scanning speeds ranged from 400 to 1400 mm/s, with the linear energy density ranging from 0.07–0.65 J/mm. In the case of the mid-temperature alloy ${NiTiHf}_{15}$, 25 single tracks were fabricated, with laser powers spanning from 80 to 240 W and scanning speeds ranging from 400 to 1200 mm/s. Finally, for the high-temperature alloy ${NiTiHf}_{20}$, 20 single tracks were fabricated, employing laser powers between 80 and 240 W and scanning speeds of 400 to 1000 mm/s. Figure 4 displays the top-view images of printed single tracks on ${NiTiHf}_{1}$, with the power ranging from 100 to 260 W at low and high scanning speeds of 400 mm/s and 1400 mm/s, respectively. Continuous single tracks were observed across various combinations of laser power and scanning speed. It was found that increasing the laser power at a constant speed resulted in wider tracks. Conversely, track width exhibited an inverse correlation with scanning speed, decreasing as the speed increased. For example, increasing the scanning speed from 400 mm/s to 1400 mm/s at a constant power of 180 W led to a decrease in single-track width. Laser printing, a rapid melting and solidification process for metal materials, involves complex phenomena, including heat transfer, fluid dynamics, and mass transfer.

These intricate physical processes directly influence the morphology, microstructure, and cracking behavior of the tracks. Cracks can be classified into longitudinal and transverse types based on their directionality and into solidification and thermal cracks based on their occurrence. These cracks can form intricate three-dimensional networks [103,104]. Surface images of single tracks reveal cracking behavior, particularly T-cracks, which are perpendicular to the laser scan direction, under all process conditions [83]. T-cracks tend to completely cross the single track and are typically formed by thermal stresses in the solid state. It is evident that T-cracks are dependent on scanning speed, with a higher number of cracks occurring as the scanning speed increases. For instance, at a lower laser power of 140 W, the number of cracks along the single tracks increases as the speed increases. These cracks likely form due to large and localized temperature gradients that generate residual stresses after the laser has passed. Higher scanning speeds result in greater thermal gradients and faster solidification, leading to higher thermal stresses and, thus, increased crack initiation [105].

**Figure 5:**
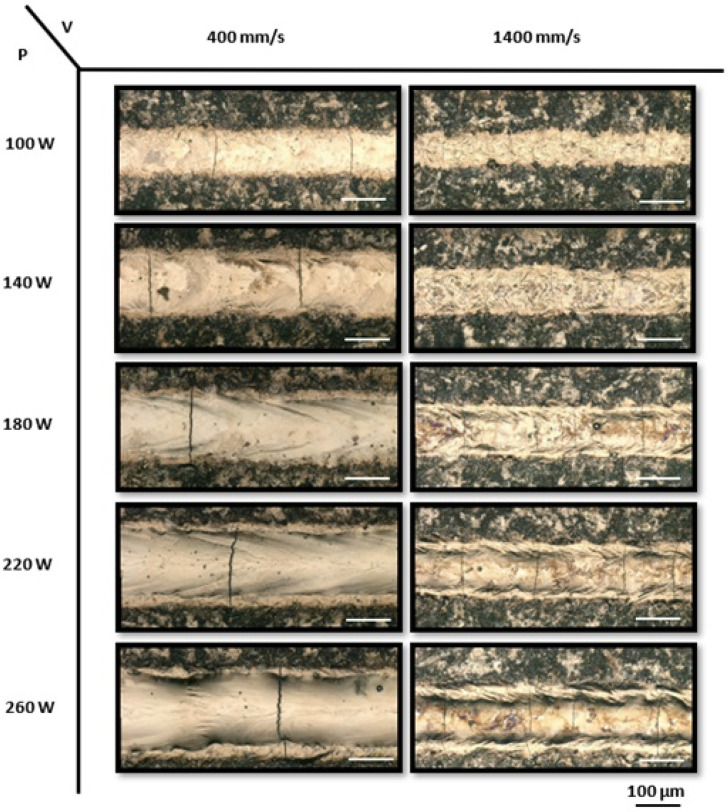
The optical micrographs of the top view of printed NiTiHf2 single tracks with laser powers ranging from 100 to 260 W and scanning speeds of 400 mm/s and 1400 mm/s.

Increasing the laser power at a constant speed results in higher heat input per unit area. This controlled increase in heat input leads to more regulated cooling rates, resulting in lower thermal gradients and reduced thermal stresses, thereby reducing the number of cracks. Compared to the single tracks printed on the ${NiTiHf}_{1}$ ingot, the single tracks printed on the ${NiTiHf}_{2}$ ingot exhibited a similar trend in the effect of processing parameters on track behavior, as shown in Figure 5. Using the same processing parameters for ${NiTiHf}_{2}$, at constant power, single tracks printed at the lowest scan speed of 400 mm/s resulted in the laser spending more time on each point. This increased heat input led to deeper penetration of the laser energy, causing fewer but deeper cracks due to significant thermal stresses and a slower cooling rate. Conversely, at higher scanning speeds, the laser spent less time on each point, resulting in lower heat input, faster cooling rates, and higher thermal gradients. This caused the formation of more and narrower cracks as the heat did not penetrate as deeply into the material.

**Figure 6:**
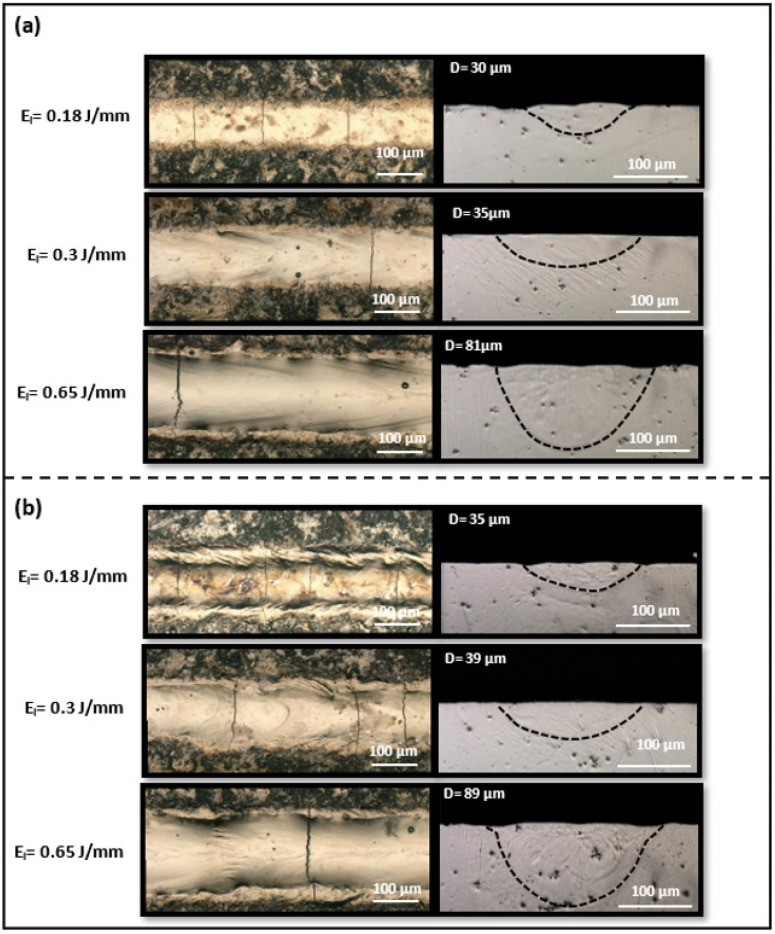
Top-view and cross-sectional images of single tracks printed at energy densities of 0.18, 0.3, and 0.65 J/mm on the (**a**) NiTi${Hf}_{1}$ ingot and (**b**) NiTi${Hf}_{2}$ ingot.

The OM images of three representative single-track morphologies on both ${NiTiHf}_{1}$ and ${NiTiHf}_{2}$ substrates are depicted in Figure 6. Figure 6a features the top views in the left panel and the cross-sectional images in the right panel of single tracks printed on ${NiTiHf}_{1}$ at three different energy densities ranging from 0.1 J/mm to 0.65 J/mm. The cross-sectional images illustrate the geometry of the melt pool, including its shape and depth, to evaluate compatibility with the melt pool geometry, typically achieved using the LPBF system with powder. Figure 6 illustrates the stable melt pools without any crack formed for single tracks fabricated across a range of energy densities, from low to high. As the energy density increases, the geometry of the melt pool changes, resulting in wider and deeper melt pools. Higher laser power or lower scanning speeds lead to higher energy input, which raises the temperature of the melt pool and extends its liquid lifetime. The increased temperature reduces the viscosity of the molten metal and enhances its wetting ability, thus producing wider and deeper melt pools [106]. Compared to the melt pools of tracks fabricated on ${NiTiHf}_{2}$ ingots (Figure 6b), the depth of the melt pools slightly increases for single tracks printed on ${NiTiHf}_{2}$ at both low and high energy densities.

**Figure 7:**
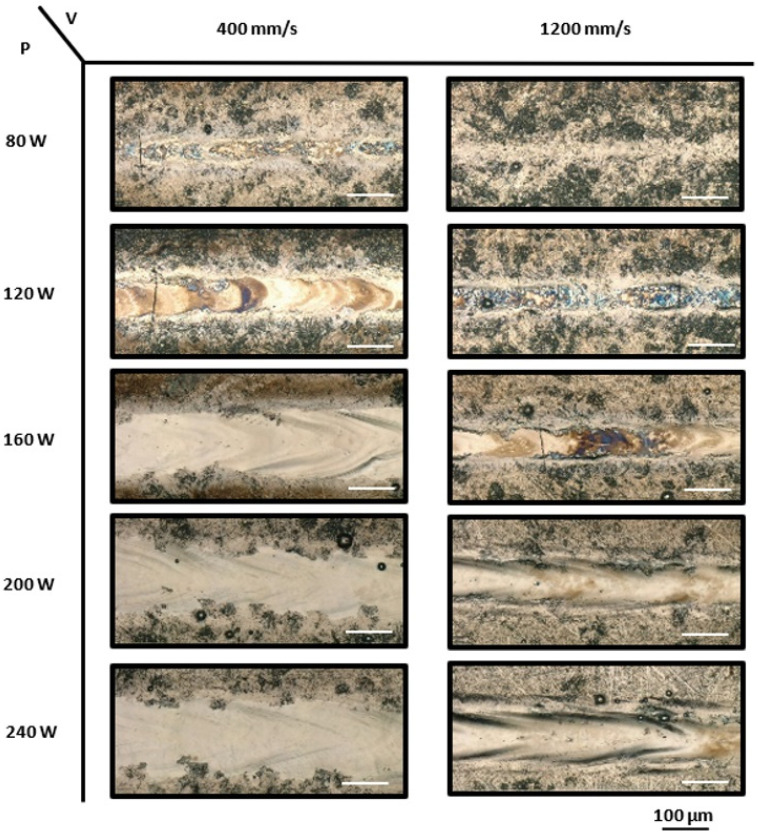
The optical micrographs of the top view of printed NiTiHf15 single tracks with laser powers ranging from 80 to 240 W and scanning speeds of 400 mm/s and 1200 mm/s.

At a low energy density of 0.18 J/mm, ${NiTiHf}_{1}$ exhibited more irregular tracks compared to ${NiTiHf}_{2}$. Additionally, more cracks were observed in ${NiTiHf}_{2}$ at low and moderate energy densities, validating the results shown in Table 3, which indicates that ${NiTiHf}_{2}$ has higher susceptibility to cracking than ${NiTiHf}_{1}$. Single tracks remelted on the ${NiTiHf}_{15}$ ingot with varied laser power ranging from 80 to 240 W and low and high scanning speeds of 400 mm/s and 1200 mm/s, respectively (Figure 7) showed a different behavior than the two previous alloys.

Four different track morphologies are identified: insufficient melting, melt tracks with cracks, stable tracks, and irregular melt tracks. With a lower laser power of 80 W and a scanning speed ranging from 400 to 1200 mm/min, the laser only marked the solid plate without causing any melting or solidification, indicating insufficient melting. It is worth noting that, except for the 80 W power setting, all laser powers ranging from 120 to 240 W, combined with scanning speeds from 400 to 1200 mm/s, produced continuous tracks. This indicates that higher laser power facilitates the formation of continuous tracks. The presence of both continuous and discontinuous tracks under various laser power and scanning speed combinations demonstrates that the morphology of the single tracks is highly dependent on the processing parameters. As the laser power increased from 80 to 120 W, T-cracks perpendicular to the laser scanning path appeared across all speed ranges, from low to high. Stable single tracks without any cracks were achieved at a laser power of 160 W and speeds ranging from 400 to 1000 mm/s; however, cracks formed at a higher speed of 1200 mm/s. These findings highlight that high heat input results in the absence of cracks, whereas tracks exhibited irregularities and waviness at higher scanning speeds (240 W and 1200 mm/s). Consequently, stable, smooth, and crack-free tracks were observed for scanning speeds of 400 to 1000 mm/s at an intermediate laser power of 160 W and at low speeds of 600 to 800 mm/s for higher laser powers of 200 to 240 W.

**Figure 8:**
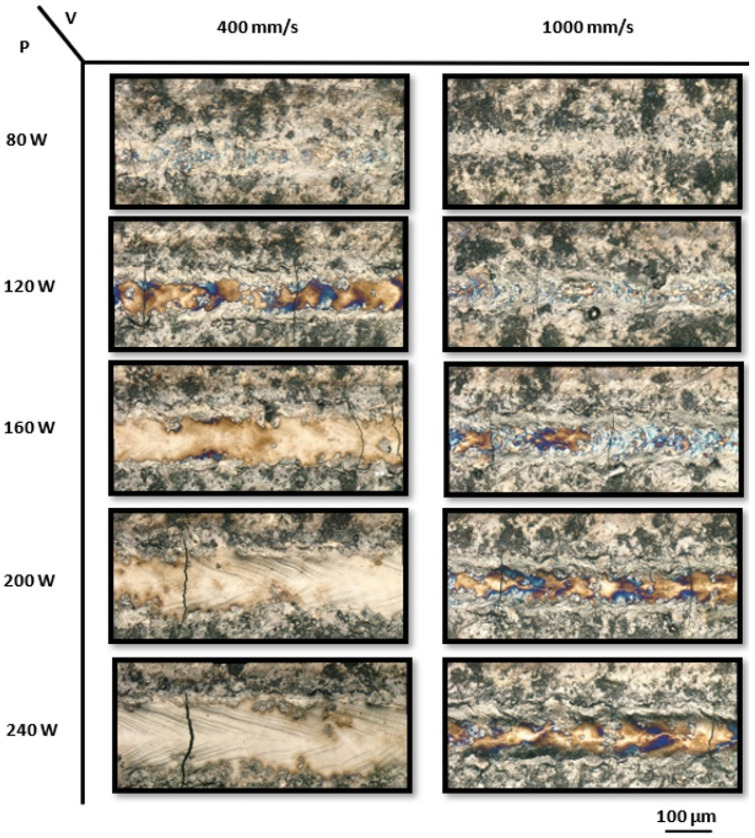
The optical micrographs of the top view of printed NiTiHf20 single tracks with laser powers ranging from 80 to 240 W and scanning speeds of 400 mm/s and 1000 mm/s.

The behavior of single tracks printed on the ${NiTiHf}_{20}$ ingot using varying laser powers ranging from 80 to 240 W, along with low and high scanning speeds of 400 mm/s and 1000 mm/s, respectively, is illustrated in Figure 8. At a lower laser power of 80 W, tracks were not observed, which was an indication of the lack of melting and solidification. After increasing the power to 120 W, discontinuous tracks were seen with clear T-cracks, which were more pronounced at the higher speed of 1000 mm/s. At a power of 160 W and the lowest speed of 400 mm/s, irregular tracks were formed with the sign of cracks; however, increasing the speed resulted in more cracks and irregularity and showed unstable tracks with the sign of cracks. Higher laser power increased the waviness and irregularities with cracks. Previous studies using ${NiTiHf}_{20}$ powder have explored the influence of processing parameters on crack formation. Nematolahi et al. [107] investigated a range of laser powers (100–250 W) and scanning speeds (200–1000 mm/s) for ${Ni}_{50.4}Ti{Hf}_{20}$. They found that while approximately one-third of the parameter sets produced crack-free samples, cracks and delamination occurred at low scanning speeds (400 mm/s) combined with high volumetric energy density (150 J/mm^2^). These defects were attributed to the interplay of high thermal gradients and insufficient bonding due to low energy input. Zhang et al. [108] also investigated ${NiTiHf}_{20}$ and identified a narrow process window for achieving porosity levels below 1% in the fabricated samples.

**Figure 9:**
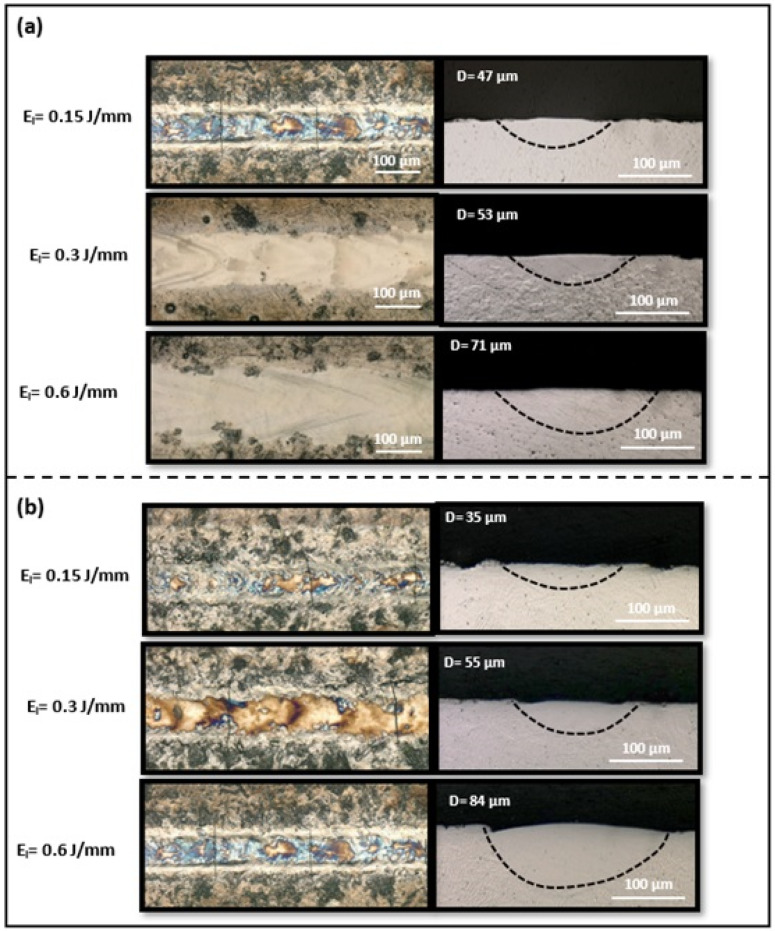
Top-view and cross-sectional images of single tracks printed at energy densities of 0.15, 0.3, and 0.6 J/mm on the (**a**) NiTiHf15 ingot and (**b**) NiTiHf20 ingot.

The optical microscopy (OM) images provided valuable insights into the morphology of single tracks printed on both ${NiTiHf}_{15}$ and ${NiTiHf}_{20}$ substrates, as depicted in Figure 9. In the left panel of Figure 9a, top-view images of the single tracks offer a detailed examination of their surface characteristics, while the right panel provides cross-sectional views, allowing for a deeper understanding of their internal structure. These observations illuminate the behavior of the melt pools formed during the printing process, particularly concerning their stability and dimensional attributes. Upon closer inspection, it becomes evident that single tracks produced on ${NiTiHf}_{15}$ substrates exhibit stable melt pools across a range of energy densities, spanning from 0.1 J/mm to 0.6 J/mm. Notably, the absence of cracks within these melt pools emphasizes their strength and structural integrity, vital for ensuring the reliability of printed components.

Furthermore, as the energy density increases, the width and depth of the melt pools experience proportional expansion, indicating a consistent response to variations in energy input. Comparing the melt pools generated on ${NiTiHf}_{20}$ substrates reveal intriguing insights into their behavior under different printing conditions. While the depth of the melt pools shows slight increases at low and high energy densities, the tracks printed at medium to higher energy levels display irregularities, characterized by waviness and the presence of cracks. Despite these deviations from the ideal morphology, the overall shape and stability of the melt pools remain largely intact, highlighting the resilience of the printing process. The findings presented in Table 3 and Table 4, which focus on the solidification temperature range and Kou’s index, provide compelling evidence that supports the observed trends in susceptibility to cracking. Notably, ${NiTiHf}_{15}$ exhibits superior resistance to cracking compared to ${NiTiHf}_{20}$, highlighting the critical role of alloy design in minimizing potential defects and ensuring the integrity of printed components. However, it should be noted that despite the higher susceptibility of NiTiHf20 to cracking, its printability may still be achievable through appropriate process optimization and parameter adjustments. Conversely, the lower susceptibility of ${NiTiHf}_{15}$ to cracking enhances its printability, making it a more attractive option for additive manufacturing applications where solidification cracking is a concern.

## 6. Conclusions

In this study, a computational analysis was conducted to evaluate the printability of four NiTiHf alloys (1%, 2%, 15%, and 20% Hf) and to assess the impact of solidification cracking on printability. The analysis employed Kou’s index and the Scheil–Gulliver model to determine alloy susceptibility to cracking and to identify the most suitable material for laser powder bed fusion (LPBF) processing. Based on the findings, the following conclusions are drawn:

- Computational Analysis Results: NiTi${Hf}_{15}$ exhibited the lowest susceptibility to cracking, as indicated by its smallest solidification temperature range and Kou’s index values. This suggests superior printability and stability under LPBF conditions.
- Cracking Tendencies: NiTi${Hf}_{2}$ and NiTi${Hf}_{20}$ showed significant cracking tendencies, whereas NiTi${Hf}_{1}$ demonstrated moderate susceptibility.
- Experimental Validation: Experimental results confirmed the computational predictions, with consistent trends observed in single-track stability and melt pool shape relative to variations in laser power and scanning speed. Higher laser power generally produced wider tracks, while increased scanning speeds heightened the likelihood of cracks.
- Material Performance: Among the alloys studied, NiTi${Hf}_{15}$ displayed exceptional uniformity and stability concerning track width, height, and shape. This advantageous melt pool geometry resulted in reduced cracking susceptibility, attributed to the intrinsic properties and compositional integrity of NiTi${Hf}_{15}$.
- Implications for Bulk Fabrication: The analysis of single-track experiments provided a comprehensive understanding of process dynamics, facilitating the prediction and control of material behavior during bulk fabrication. The insights gained were critical in refining laser parameters to achieve uniform layer deposition, minimize defects, and ensure consistent material properties.
- Future Research: These findings will be instrumental in optimizing LPBF parameters for NiTiHf alloys once the powders become available, ensuring the efficient and reliable production of high-temperature shape memory components.

## Figures and Tables

**Table 1:** Chemical composition of NiTiHf with different compositions (1, 2, 15, and 20 at. %).

Alloys	Ni (at. %)	Ti (at. %)	Hf (at. %)	Zr (at. %)	C (wt. %)	N (wt. %)	O (wt. %)
Ni_50.3_TiHf_1_	50.5	47.8	1	0.04	0.149	0.0005	0.0223
Ni_50.3_TiHf_2_	50.3	47.1	1.9	0.07	0.126	0.0006	0.0189
Ni_50_TiHf_15_	49.92	34.98	14.39	0.71	0.056	0.107	0.106
Ni_50.3_TiHf_20_	50.35	29.69	19.81	0.15	0.0077	0.00245	0.04815

**Table 2:** The calculated liquidus, solidus, and solidification range of NiTiHf ingots.

Composition	Liquidus (°C)	Solidus (°C)	Solidification Range (°C)
NiTiHf_1_	1300.14	1073.05	227.09
NiTiHf_2_	1289.57	958.33	331.24
NiTiHf_15_	1146.44	1091.35	55.09
NiTiHf_20_	1097.78	841.86	255.92

**Table 3:** The crack susceptibility prediction based on Kou’s index.

Composition	NiTiHf_1_	NiTiHf_2_	NiTiHf_15_	NiTiHf_20_
Kou’s index (×10^3^)	0.9084	0.4394	0.0058	1.8826

**Table 4:** The normalized cracking index for the NiTiHf alloys.

Composition	NiTiHf_1_	NiTiHf_2_	NiTiHf_15_	NiTiHf_20_
STR *	0.686	1	0.166	0.773
SC (Kou) **	0.482	0.233	0.003	1

* Non-equilibrium solidification index for the Hf ingots. ** Steepness criteria (SC).

**Table 5:** The phase transformation temperatures of four NiTiHf alloys with an Hf content of 1, 2, 15, and 20 at. %.

Alloys	$M_{f}$ (°C)	$M_{s}$ (°C)	$A_{s}$ (°C)	$A_{f}$ (°C)	$A_{f}$ (°C) − $M_{s}$ (°C)
Ni_50.3_TiHf_1_	−60	18	−79	24	6
Ni_50.3_TiHf_2_	−100	−51	−48	−10	−41
Ni_50_TiHf_15_	73.34	93.55	122.79	141.04	47.49
Ni_50.3_TiHf_20_	155.21	193.25	203.85	226.13	32.88

**Table 6:** The process parameter (P, V) set for the NiTiHf single-track process.

Alloys	Power (W)	Speed (mm/s)	E_l_ (J/mm)
NiTiHf_1_	100, 140, 180, 220, 260	400, 600, 800, 1000, 1200, 1400	0.07–0.65
NiTiHf_2_	100, 140, 180, 220, 260	400, 600, 800, 1000, 1200, 1400	0.07–0.65
NiTiHf_15_	80, 120, 160, 200, 240	400, 600, 800, 1000, 1200	0.07–0.6
NiTiHf_20_	80, 120, 160, 200, 240	400, 600, 800, 1000	0.07–0.5

## Data Availability

The original contributions presented in the study are included in the article, further inquiries can be directed to the corresponding author.
